# Effects of EMG-based robot for upper extremity rehabilitation on post-stroke patients: a systematic review and meta-analysis

**DOI:** 10.3389/fphys.2023.1172958

**Published:** 2023-05-03

**Authors:** Yunxia Huo, Xiaohan Wang, Weihua Zhao, Huijing Hu, Le Li

**Affiliations:** ^1^ Institute of Medical Research, Northwestern Polytechnical University, Xi’an, China; ^2^ Research & Development Institute of Northwestern Polytechnical University in Shenzhen, Shenzhen, China; ^3^ Northwestern Polytechnical University Hospital, Xi’an, China

**Keywords:** EMG-based robot, stroke, upper extremity, meta-analysis, review

## Abstract

**Objective:** A growing body of research shows the promise and efficacy of EMG-based robot interventions in improving the motor function in stroke survivors. However, it is still controversial whether the effect of EMG-based robot is more effective than conventional therapies. This study focused on the effects of EMG-based robot on upper limb motor control, spasticity and activity limitation in stroke survivors compared with conventional rehabilitation techniques.

**Methods:** We searched electronic databases for relevant randomized controlled trials. Outcomes included Fugl-Meyer assessment scale (FMA), Modified Ashworth Scale (MAS), and activity level.

**Result:** Thirteen studies with 330 subjects were included. The results showed that the outcomes post intervention was significantly improved in the EMG-based robot group. Results from subgroup analyses further revealed that the efficacy of the treatment was better in patients in the subacute stage, those who received a total treatment time of less than 1000 min, and those who received EMG-based robotic therapy combined with electrical stimulation (ES).

**Conclusion:** The effect of EMG-based robot is superior to conventional therapies in terms of improving upper extremity motor control, spasticity and activity limitation. Further research should explore optimal parameters of EMG-based robot therapy and its long-term effects on upper limb function in post-stroke patients.

**Systematic Review Registration:** https://www.crd.york.ac.uk/PROSPERO/; Identifier: 387070.

## 1 Introduction

Stroke is a prevalent neurological dysfunction syndrome characterized by high incidence, mortality and disability rate (Winstein et al., 2016). Upper limb disorders are present in 85% of stroke survivors, with motor dysfunction still affecting 55%–75% of patients 3–6 months after onset (Parker et al., 1986; Feys et al., 1998). The residual upper limb dysfunction hugely impacts the ability of post-stroke patients to live and work independently, leading to reduced quality of life and a burden on patients’ families and society (Micera et al., 2020). Therefore, there is an urgent need to promote upper extremity function in post-stroke patients. However, the need for effective rehabilitation techniques for upper limb in stroke survivors remains largely unmet. Thus, it is of great significance to develop effective and positive rehabilitation methods for the upper limb rehabilitation of stroke survivors.

Conventional rehabilitation techniques, such as constraint-induced movement therapy (CIMT), physical therapy (PT) and occupational therapy (OT) (Pollock et al., 2014; Corbetta et al., 2015), have been adopted to assist upper limb rehabilitation. These techniques require patients to perform full or partial-assisted movements under the supervision of therapists. Other rehabilitation methods such as electrical stimulation (ES) and robots can provide repetitive, high-intensity training and also have benefit to reduce the physical stress of rehabilitation staff (Doucet et al., 2012; Zhang et al., 2017; Wang et al., 2021). However, a lack of real-time feedback from patients and excessive electrical stimulation may impede the efficacy of repetitive, high-intensity training and, in certain instances, induce muscle fatigue, thereby hindering the facilitation of motor function recovery (Chae et al., 2002).

Electromyography (EMG) have been utilized to control electrical stimulations (Hu et al., 2010; Rong et al., 2015; Rong et al., 2017; Nam et al., 2022) and powered exoskeletal devices (Rosen et al., 2001; Cheng et al., 2003; Dipietro et al., 2005; Ferris et al., 2005; Song et al., 2008) and trigger robot-assisted training to provide movement assistance (Zhuang et al., 2021). The EMG-based robot is one of the novel techniques designed for maximizing the involvement of voluntary efforts during post-stroke training. Unlike traditional robot-assisted training, the EMG-based robot can detect residual EMG signals of the affected limb in real time and integrate the participants’ voluntary motor intention represented by the EMG signal from the residual muscles into training (Song et al., 2008; Rong et al., 2015; Chen et al., 2022). Once the EMG signals reach a specific threshold, the robot-assisted training will be activated, assisting the patients to complete a desired movement. The EMG-based robot could increase the interaction between participants and machines, potentially enhancing the effect of robot-assisted training, and reducing the pressure on medical staff. Studies have found that the EMG-based robot improved the Fugl-Meyer assessment (FMA) score and spasticity of the upper extremity in post-stroke patients when compared with conventional therapy, (Stein et al., 2007; Song et al., 2008; Hu et al., 2015; Nam et al., 2017). However, Chen et al., (Chen et al., 2022), and Page et al., (Stein et al., 2007; Page et al., 2013; Page et al., 2020), respectively found the efficacy of the EMG-based robot was not superior to task-oriented training and conventional hands-on manual therapy. Therefore, it remains unclear whether the effect of the EMG-based robot is superior to conventional therapies on upper limb function of stroke survivors.

To date, there is a lack of meta-analyses summarizing whether EMG-based robot training is superior to conventional treatment. Therefore, the objectives of this meta-analysis are twofold: 1) to determine the superiority of EMG-based robot therapy over conventional therapy, and 2) to analyze the effectiveness of different treatment options.

## 2 Methods

This review was on the basis of the Preferred Reporting Items for Systematic Reviews and Meta-Analyses (PRISMA) (Moher et al., 2009).

### 2.1 Search strategy

We searched studies published before 20 November2022 in these electronic databases: Embase, Scopus, PubMed (MEDLINE), Cochrane library and Web of science. The PICOS (participant, intervention, comparison, outcome and study design) framework was used to research. When determined the systematic keywords to retrieve, we only used P and I to avoid missing crucial articles. The mesh terms used in Embase were “Cerebrovascular Accident” (Participants), “Electromyography” AND “Robotics” (Intervention), and in other databases were related to “Stroke” (Participants), “Electromyography” AND “Robot” (Intervention). Detailed search strategy for those databases could be found in Supplementary Material.

### 2.2 Inclusion and exclusion criteria

The following was the inclusion criteria: 1) Participants: patients who have been suffering stroke; 2) Intervention: EMG-based robot therapies, including EMG-driven robot, smart rehabilitation systems with EMG, and electromechanical orthosis; 3) Outcomes: measures of upper extremity motor function, spasticity and activity limitation; 4) Study design: randomized controlled, cross-over clinical trials; 5) articles published in English.

The following was the exclusion criteria: 1) studies not focusing on post-stroke patients; 2) no EMG-based robot system described; 3) studies targeting the low limb functions of stroke; 4) case reports; 5) review articles; 6) studies without the full text. Two reviewers assessed each article for inclusion independently. When an identity of views was not reached between the two reviewers, the ratings were discussed among all co-authors until a consensus was reached.

### 2.3 Outcome measures

Based on the International Classification of Functioning Disability and Health model (ICF) (Reis et al., 2021), we classified outcomes into a) motor control of upper limb, b) muscle tension, and c) activity capacity. The above three aspects were respectively evaluated by Fugl-Meyer Assessment Scale (FMA), modified Ashworth Scale (MAS) and activity limitation (e.g., Box and Blocks Test [BBT], Nine Hole Peg Test, Jebsen-Taylor Hand Function Test, Action Research Arm Test [ARAT]) (Veerbeek et al., 2017; Reis et al., 2021).

### 2.4 Data extraction

Two authors analyzed the abstracts and contents of each article carefully and extracted the data systematically. If there was a disagreement, it would be resolved by consultation with a third author. We extracted data on the following two aspects: 1) the basic information of the study, including the type of study, demographic characteristics of the subjects, outcomes measures, and 2) information on the EMG-based robot systems, including robot types, parameters of intervention. We contacted authors for original data when partial data were not available.

### 2.5 Risk of bias assessments

We used the Cochrane risk of bias assessment tool (Higgins and Altman, 2008) to assess each study by two independent authors. This tool contains six items, selection bias, performance bias, detection bias, attrition bias, reporting bias, and other biases (Higgins et al., 2011). Disagreements were resolved by consultation with a third reviewer when necessary.

### 2.6 Statistical analysis

Review Manager 5.4 (The Nordic Cochrane Centre, The Cochrane Collaboration, Copenhagen, Denmark) was used to analysis. We compared variation in effect sizes on the outcome measures of upper extremity function between the experimental group (EG, using EMG-based robot) and control group (CG, using conventional therapies) before and after intervention.

Then, we used mean difference (MD) or standardized mean difference (SMD) and 95% confidence intervals (CIs) to calculate the pooled effects of outcome measures. In addition, we evaluated heterogeneity by examining forest plots, chi^2^ test and I^2^ statistic were used to assess the heterogeneity between RCTs. I^2^ values range from 0% to 100%, and are considered low at <25%, modest at 25%–50%, and high at >50%, and the t-statistic is being used for the degrees of freedom in the random effects analysis, when the number of studies is small (e.g., <10). Subgroup analyses, meta-regression and sensitivity analysis were computed to evaluate heterogeneity.

## 3 Results

### 3.1 Study screening

**FIGURE 1:**
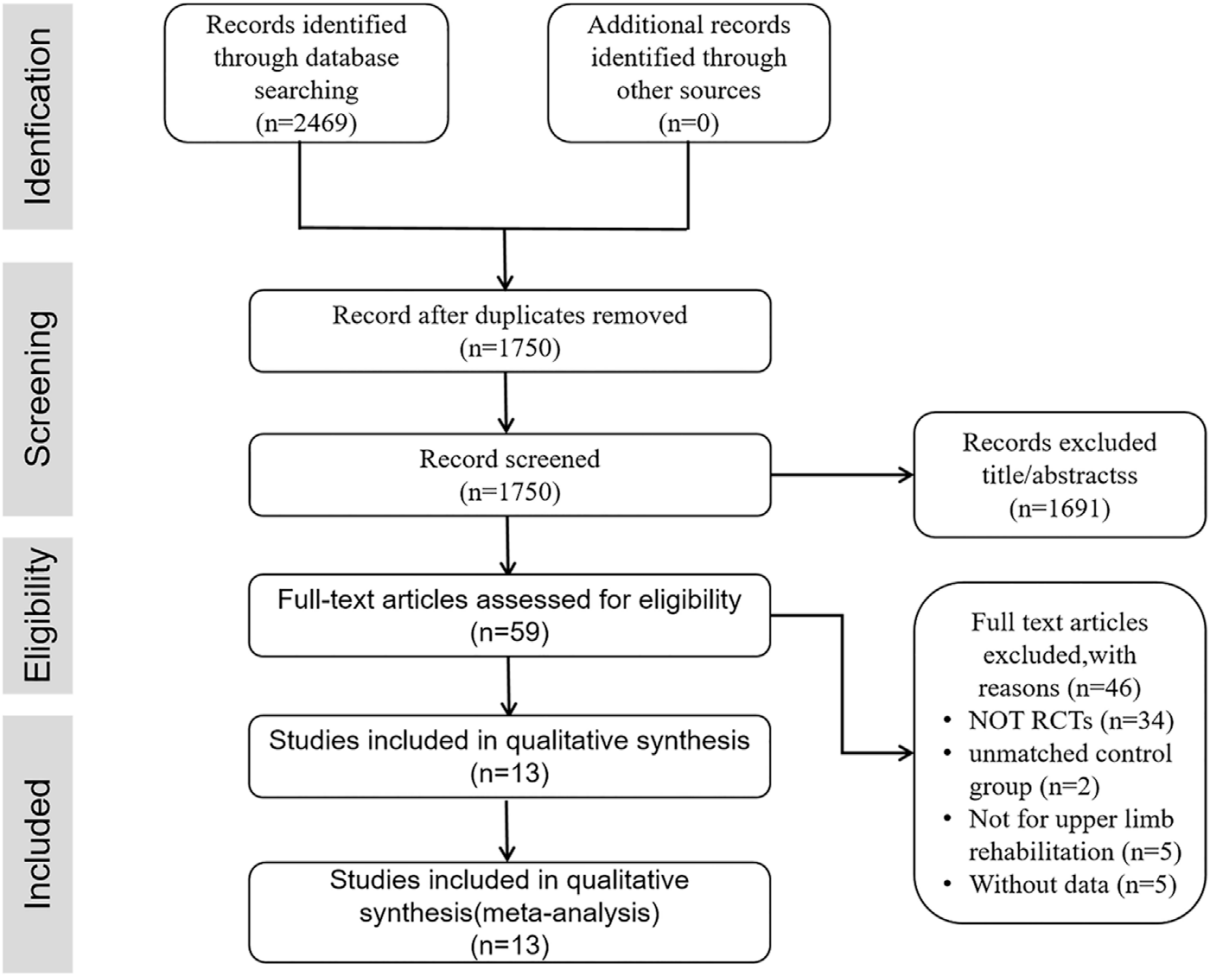
The PRISMA flow diagram.

Figure 1 shows the details of the whole selection process. A total of 2,469 articles were retrieved after searching the databases. And 1750 articles were screened after removing 719 duplicates, of which 1,691 articles were excluded according to the PICOS principle in titles and abstracts. The rest of 59 studies were submit to full-text checking, of which 13 studies were included for meta-analysis.

### 3.2 Quality of the included studies

**FIGURE 2:**
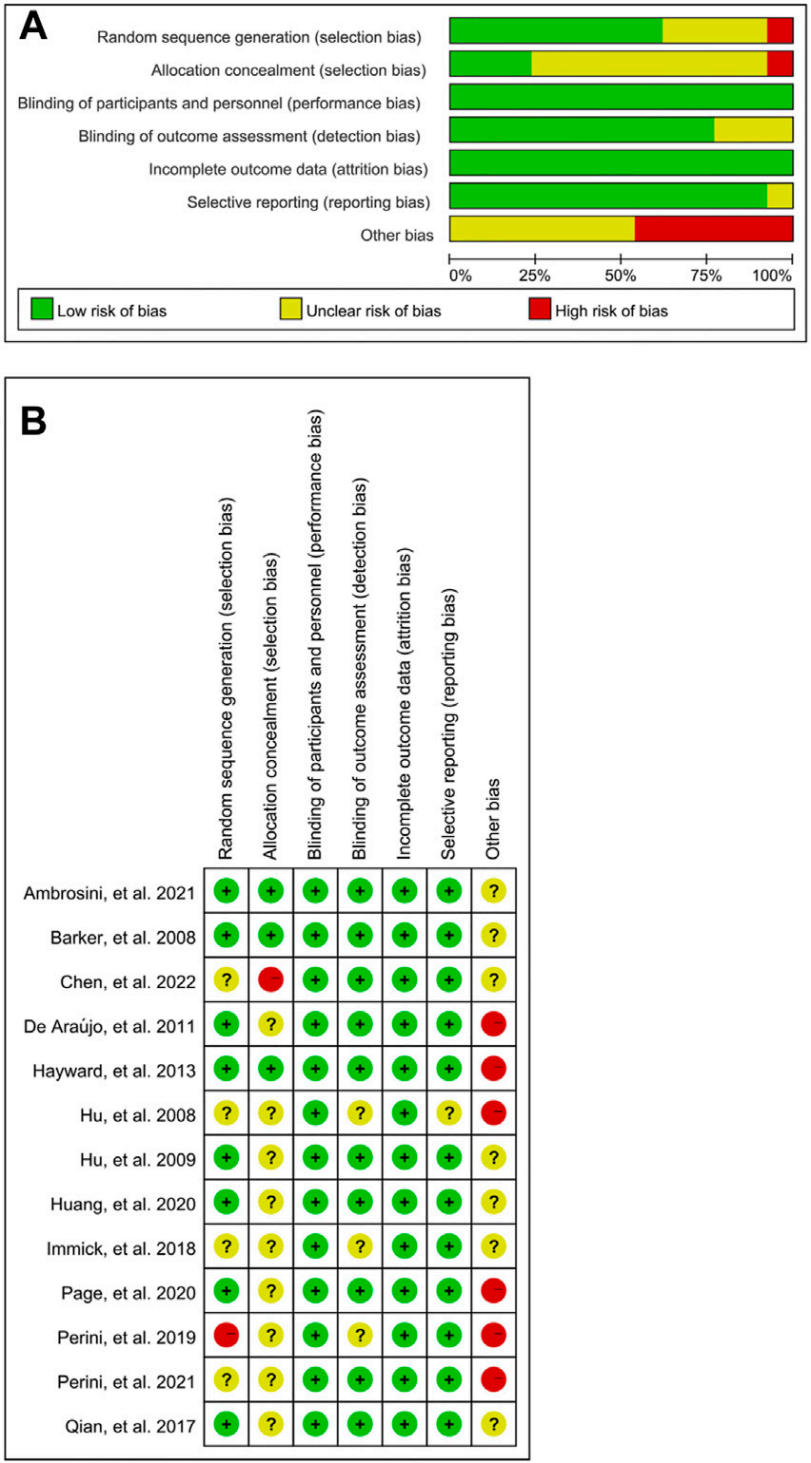
Risk of bias in the systematic review. Bias of the included articles is relatively low and stable. **(A)** Risk of bias for all included studies. **(B)** Risk of bias item for each included study.

Risk of bias for included 13 studies was assessed by two reviewers independently. The results were shown in Figure 2, and sensitive analysis indicated that the results appeared to be stable.

### 3.3 Study characteristics

Table 1 shows the details of basic information of the included trials. In total, this meta-analysis included 330 subjects (EG, *n* = 175; CG, *n* = 155). One study was extracted two experimental groups (Page et al., 2020). For randomized cross-over trial (Chen et al., 2022), data before crossover were extracted. The study with fewest number of participants was carried out by Hayward (*n* = 8, EG:4, CG:4) (Hayward et al., 2013). The mean duration of post-stroke ranged from 0.87 months to 8.3 years. Most studies concentrated on stroke rehabilitation in chronic stage.

**TABLE 1:** Basic information about included studies.

Study	Intervention EG vs. CG	Participants	Age(years)	Post-stroke duration(months)	Intervention time setting			Outcome measures
			Mean ± SD	Mean ± SD	Session duration (minutes)	Frequency (weeks)	Weeks	
Ambrosini et al., 2021	EMG-driven FES robot vs. Conventional Therapies	EG:36	EG:60.9 (13.7)	EG:2.11 (1.96)	30	3	9	ARAT, BBT
		CG:36	CG:67.8 (12.2)	CG:2.07 (2.56)				
Barker et al. (2008)	Smart rehabilitation system and stim vs. smart rehabilitation system alone	EG:10	EG:61 (16)	EG:60 (58.8)	60	3	4	MAS
		CG;13	CG:67 (8)	CG:40.8 (31.2)				
Chen et al. (2022)	EMG-driven robot vs. Task Oriented	EG:14	EG:54.58 (10.98)	EG:37.07 (34.39)	60	3	8	FMA, ARAR
		CG:10	CG:64.98 (8.22)	CG:59.8 (43.34)				
De Araújo et al., 2011	Electromechanical orthosis vs. Conventional Therapies	EG:6	EG:42.83 (14.04)	EG:21.67 (11.83)	50	3	8	FMA, MAS
		CG:6	CG:52.67 (17.84)	CG:19.00 (11.01)				
Hayward et al. (2013)	Smart rehabilitation system and stim vs. smart rehabilitation system alone	EG:4	EG:69 (10)	EG:1.53 (0.4)	60	5	4	MAS
		CG:4	CG:56 (24)	CG:0.87 (0.2)				
Hu et al. (2009)	EMG-Driven robot vs. passive device	EG:15	EG:49.2 (14.7)	EG:56.4 (50.4)	36.5	3–5	4–7	FMA, MAS, ARAT
		CG:12	CG:53.3 (10.4)	CG:61.2 (49.2)				
Hu et al. (2009)	EMG-Driven robot vs. passive device	EG:5	EG:50.2 (10.2)	EG:12	36.5	3–5	4–6	FMA, MAS
		CG:5	CG:50.2 (10.2)	CG:12				
Huang et al., 2020	EMG-Driven NMES-robot vs. robot	EG:15	EG:57.33 (9.19)	EG:99.24 (51.84)	60	3–5	4–7	FMA, MAS, ARAT
		CG:15	CG:60.07 (6.88)	CG:74.4 (40.92)				
Immick et al., 2018	EMG-driven robot vs.Conventional Therapies	EG:19	EG:59.0 (15.9)	EG:2.14 (2.2)	30	3	9	ARAT, BBT
		CG:20	CG:67.7 (12.1)	CG:2.65 (3.13)				
Page et al. (2020)	Myomo vs. RTP	EG:14	EG:55.79 (9.25)	EG: N	30	3	8	FMA
		CG: 5	CG:57.22 (7.68)	CG: N				
Page et al. (2020)	Myomo + RTP vs. RTP	EG:8	EG:52.89 (11.38)	EG: N	30	3	8	FMA
		CG:5	CG:57.22 (7.68)	CG: N				
Perini et al., 2019	MeCFES + robot vs. Conventional Therapies	EG:6	EG:65.5 (23.1)	EG:20 (10.3)	45	5	4	FMA, ARAT
		CG:5	CG:65.5 (23.1)	CG:20 (10.3)				
Perini et al., 2021	MeCFES + robot vs. Task Oriented	EG:9	EG:58.7 (20.6)	EG:35.3 (44.5)	90	5	4	FMA, BBT
		CG:9	CG:61.4(9)	CG: 42 (44.7)				
Qian et al. (2017)	EMG-Driven NMES-robot vs. Conventional Therapies	EG:14	EG:54.6 (11.3)	EG:0.83	40	5	4	FMA, MAS, ARAT
		CG:10	CG:64.6 (3.43)	CG: 0.46				

EG, experimental group; CG, control group; Myomo, myoelectric device; RTP, repetitive, task-specific practice; NMES, neuromuscular electrical Stimulation; MeCFES, myoelectric control functional electrical stimulation; FMA, fugl-mayer assessment; MAS, modified ashworth scale; ARAT, action research arm test; BBT, box and block test.

In terms of setting EMG-based robot intervention parameters, the session duration of EMG-based robot ranged from 30 min to 90 min, and the average was 47 min. Eight studies set a session less than 60 min. Three studies (Barker et al., 2008; Hayward et al., 2013; Chen et al., 2022) set a session duration of 60 min, and others were more than 60 min. The average frequency of intervention was 3.79 times/week, and most studies focused on a frequency of 3 times/week. The average intervention period was 6.04 weeks, and most studies continued 4 weeks.

According to the statistics, the effect of the intervention on participants was mainly confirmed by measuring subjects’ upper limb motor function, spasticity and activity limitation. The FMA, MAS and assessment for activity level, such as ARAT, BBT were the most commonly used scales.

### 3.4 Synthesis of results

#### 3.4.1 Effect of EMG-based robot therapy compared with control group

**FIGURE 3:**
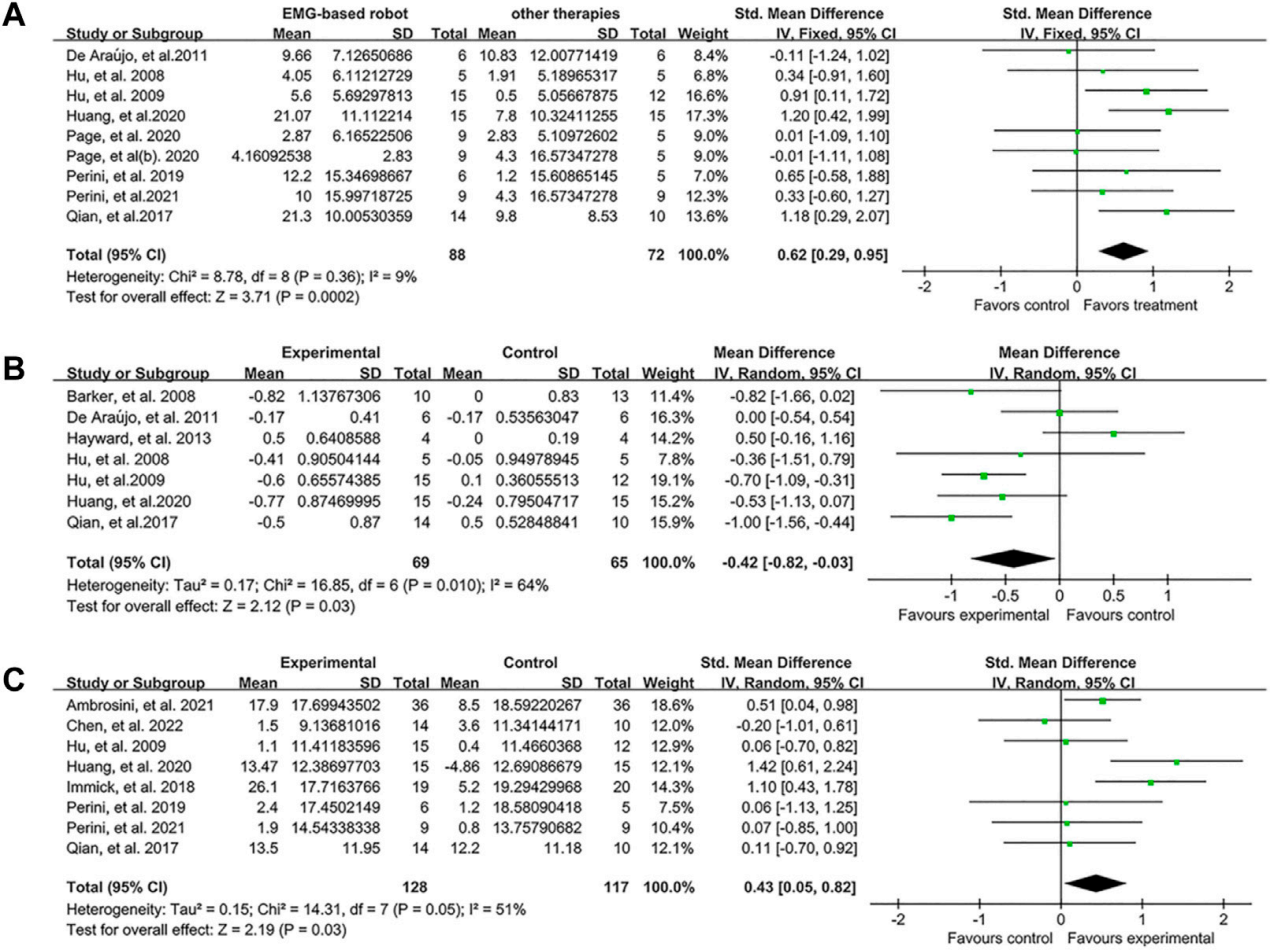
Forest plot analysis of the effect of EMG-based robot therapy vs. conventional therapies. **(A)** Forest plot analysis of the effect of EMG-based robot therapy vs. conventional therapies on FMA. **(B)** Forest plot analysis of the effect of EMG-based robot therapy vs. conventional therapies on MAS. **(C)** Forest plot analysis of the effect of EMG-based robot therapy vs. conventional therapies on activity limitation.

**FIGURE 4:**
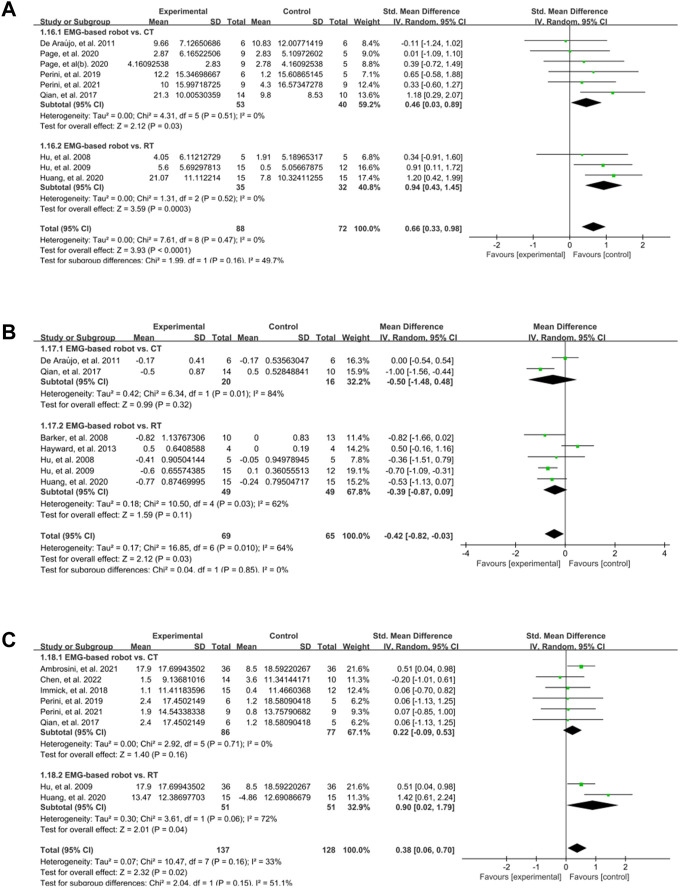
A subgroup analysis of the effect of EMG-based robot vs. different control groups on outcomes. **(A)** Forest plot analysis of the effects of different control groups on FMA. **(B)** Forest plot analysis of the effects of different control groups on MAS. **(C)** Forest plot analysis of the effects of different control groups on activity limitation.

In the subgroup analyses, the EMG-based robot, non EMG-based robot (i.e., conventional rehabilitation robot such as InMotion), and conventional (non-robotic) rehabilitation were compared for the improvement of motor control, spasticity and activity limitation post intervention. Nine studies measured the effect of EMG-based robot on FMA. The results showed that the score of FMA was significantly increased (SMD:0.62, 95% CI:0.29 to 0.95) (Figure 3A). Seven studies focused on MAS, and the results showed MAS changed significantly (MD: −0.42, 95% CI: −0.82 to −0.03) (Figure 3B). Eight studies aimed at activity limitation, the results showed the activity level also improved notably (SMD:0.43, 95% CI:0.05 to 0.82) (Figure 3C). The results of subgroup analyses showed that the effect of EMG-based robot in motor control was superior to both conventional therapies (SMD:0.46, 95% CI:0.03 to 0.89) and robotic therapies (SMD:0.94, 95% CI:0.43 to 1.45) (Figure 4A). In terms of spasticity, no obvious advantage was found for the EMG-based robot over conventional treatment (Figure 4B). The EMG-based robot was more effective than the robotic therapies in the activity limitation (SMD:0.90, 95% CI:0.02 to 1.79) (Figure 4C). However, there is high heterogeneity in the outcome of MAS and activity limitation. The results of meta-regression (Supplementary Figure A1) and sensitivity analysis (Supplementary Figure A2) showed that the sample size contributed to the outcome of MAS and the duration of disease contributed to activity limitation.

#### 3.4.2 The total time of training

**FIGURE 5:**
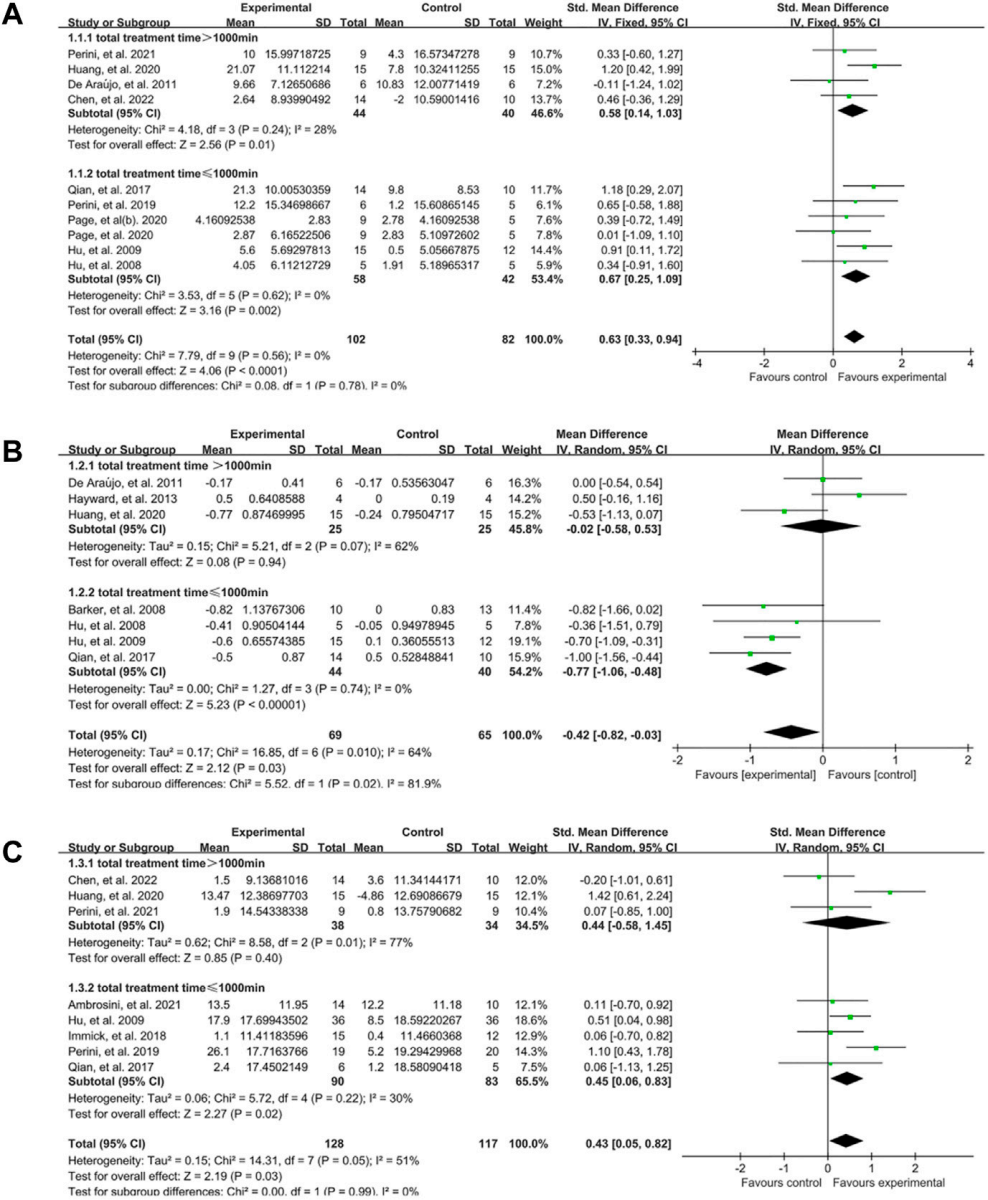
A subgroup analysis of the effect of EMG-based robot with different total training time vs. conventional therapies on outcomes. **(A)** Forest plot analysis of the effects of different total time of training on FMA. **(B)** Forest plot analysis of the effects of different total time of training on MAS. **(C)** Forest plot analysis of the effects of different total time of training on activity limitation.

As described in previous studies (Wang et al., 2021; Zhang et al., 2022), the amount of intervention was estimated by total time. We discovered that there was a significant difference in the upper limb motor function in both subgroups at the end of treatment between EMG-based robot therapy and conventional therapies, and the effect size was lager in subgroup with total training time ≤1000 min (SMD:0.67, 95% CI:0.25–1.09) than subset with total time >1000 min (SMD:0.58, 95% CI:0.14–1.03) (Figure 5A). In terms of spasticity, subgroup with total time ≤1000 min had a significant difference between EG and CG (MD: −0.77, 95% CI: −1.06 to −0.48), but no significant difference in the subset with total time >1000 min (MD: −0.02, 95% CI: −0.58 to 0.53) (Figure 5B). Activity limitation also changed significantly in subgroup with total time ≤1000 min (SMD:0.45, 95% CI:0.06–0.83), rather than subgroup with total time >1000 min (SMD:0.44, 95% CI: −0.58–1.45) (Figure 5C).

#### 3.4.3 The intervention mode

**FIGURE 6:**
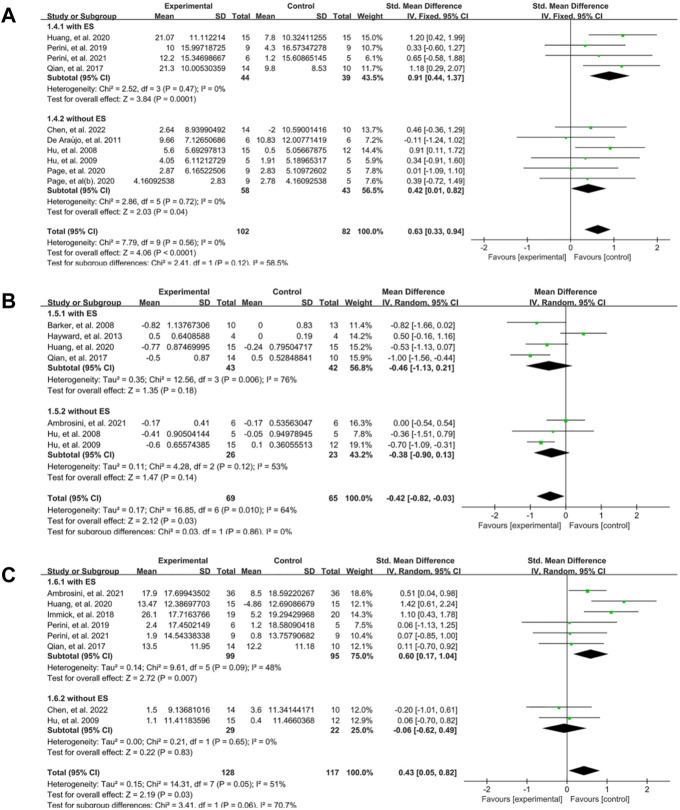
A subgroup analysis of the effect of different EMG-based robots vs. conventional therapies on outcomes. **(A)** Forest plot analysis of the effects of different intervention mode on FMA. **(B)** Forest plot analysis of the effects of different intervention mode on MAS. **(C)** Forest plot analysis of the effects of different intervention mode on activity limitation.

The intervention modes provided by EMG-based robot included EMG-driven robot with electrical stimulation (ES) and the type without ES. Subgroup analysis showed that the robot type with ES (SMD: 0.91, 95% CI:0.44–1.37) had larger effect size on the FMA than the type without ES (SMD: 0.42, 95% CI: 0.01–0.82) at the end of treatment (Figure 6A). The outcome of MAS at the end of treatment showed no significant difference between EG and CG in both subgroup (Figure 6B). In terms of the activity limitation, we found that the subgroup with ES (SMD:0.60, 95% CI:0.17–1.04) was better than the subgroup without ES (SMD: −0.06, 95% CI: −0.62–0.49) (Figure 6C).

#### 3.4.4 Stage of stroke

**FIGURE 7:**
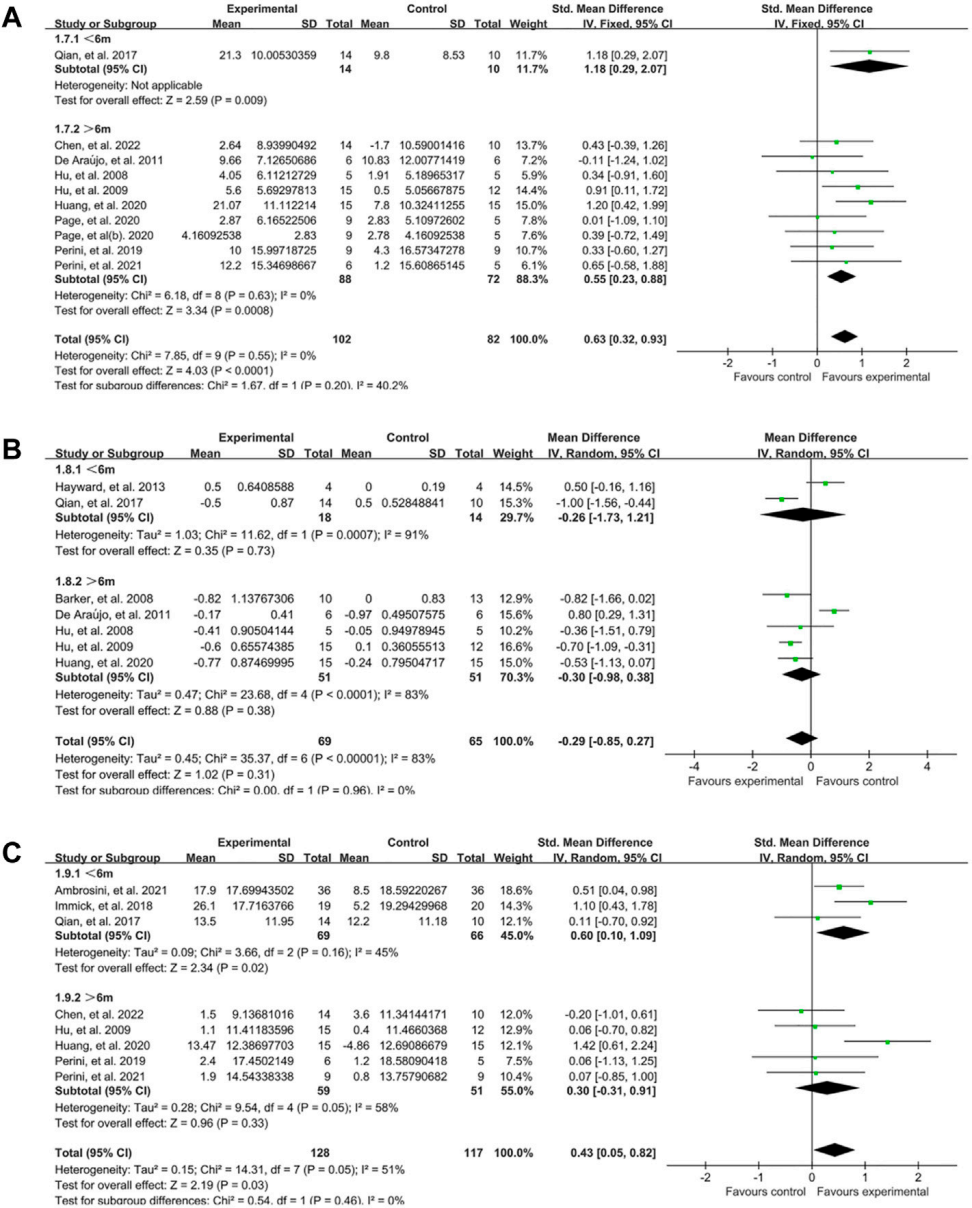
A subgroup analysis of the effect of EMG-based robot for different stage of stroke. **(A)** Forest plot analysis of the effects of different stage of stroke on FMA. **(B)** Forest plot analysis of the effects of stage of stroke on MAS. **(C)** Forest plot analysis of the effects of stage of stroke on activity limitation.

The stage of stroke was evaluated according to the duration after the onset time, and the participants were classified into subacute (≤6m) and chronic (>6m) group (Wang et al., 2021). For upper limb motor function, most studies focused on chronic stage and just one study designed by Qian, et al. (Qian et al., 2017) was in subacute stage. Analysis showed that FMA score was significantly changed in chronic group (SMD:0.55, 95% CI:0.23–0.88) and subacute group (SMD:1.18, 95% CI:0.29–2.07) (Figure 7A). For spasticity, there were no significant difference between two groups (Figure 7B). When comes to the activity limitation, the result showed the effect was better in subacute group (SMD:0.60, 95% CI:0.10–1.09) than chronic group (SMD:0.30, 95% CI: −0.31–0.91) (Figure 7C).

## 4 Discussion

In the present meta-analysis, a total of 12 RCTs and one cross-over clinical trial were analyzed, which included a total of 330 subjects (EG, *n* = 175; CG, *n* = 155). The results indicated that EMG-based robot was more effective than conventional therapies, including physical therapy, occupational therapy, passive training, and conventional robotic therapy. Results from subgroup analyses further revealed that the efficacy of the treatment was better in patients in the subacute stage, those who received a total treatment time of less than 1000 min, and those who received electromyography (EMG)-based robotic therapy combined with electrical stimulation (ES). These findings suggest that EMG-based robot therapy may be considered a promising rehabilitation method to improve upper limb dysfunction in post-stroke patients.

### 4.1 Effect of EMG-based robot

The findings presented in Figures 3, 4 demonstrate that EMG-based robot therapy can significantly enhance upper limb motor function, reduce spasticity, and improve activity level in post-stroke patients, as compared to the robotic therapies and conventional therapies. Following a stroke occurrence, the injured brain undergoes a reorganization process that involves recruiting pathways homologous to the damaged regions in function but distinct in anatomy, synaptogenesis, dendritic arborization, and reinforcing synaptic connections (Rossini et al., 2003). Such adaptive brain changes seem to be closely related to rehabilitation and motor training, contributing to the improvement of functional outcomes (Chen et al., 2018; Sampaio-Baptista et al., 2018; Sheng et al., 2022). High-intensity repetitive training is one of the principles of post-stroke rehabilitation (Langhorne et al., 2011). Although there are no clear guidelines for the optimal level of practice, it is widely accepted that more intensive training is beneficial. Robot-assisted therapy can provide patients with repetitive, high-intensity training and improve their motor function (Zhang et al., 2017; Kim et al., 2020; Wang et al., 2021; Yeung et al., 2021). The possible mechanism involves enhancing neural plasticity and neuronal activity to facilitate neuroplasticity change (Chen et al., 2019; Xing and Bai, 2020). According to the Hebbian learning rule, the connections between neurons are strengthened when neurons are simultaneously active (that is, long-term potentiation) (Orbach, 1998). In addition, signals from EMG-based robot can reflect the voluntary movement intention of patients in real time (Rong et al., 2015; Zhuang et al., 2021), which may increase active patient participation and promote interaction between humans and machines. Recording of muscle activity by EMG has proved to be helpful to explore the activity state of muscle tissue and the control mechanism of the nervous system under different task states after stroke (Ma et al., 2017; Chen et al., 2018), and then be useful for evaluating central and peripheral determinants of motor dysfunction which facilitates the understanding of mechanism behind rehabilitation intervention (Sheng et al., 2022; Li et al., 2023). Voluntary motor intention is crucial in the rehabilitation of motor function after stroke (Yang et al., 2022). Rehabilitation training that incorporates participants’ intention input is more effective which further facilitate active participation (Hu et al., 2009; Hu et al., 2021; Zhang et al., 2022) and interactive control (Cozens, 1999). The formation of a sensorimotor cycle by the voluntary intention output and the afferent sensor might facilitate motor relearning in post-stroke (Cauraugh et al., 2000). Furthermore, the effectiveness of repeated training increased substantially with the increase interaction between patients and machine (Hu et al., 2009; Hu et al., 2021). In general, the results of current study support the clinical application of EMG-based robot therapy in improving upper limb dysfunction in patients after stroke. However, more investigation is needed to reveal the relation between the changes of brain function and improvements of neuromuscular systems (i.e., by using cortical-muscular coherence technique) during the EMG-based robot training. This might be helpful for the understanding of the potential mechanism related to neuroplasticity in post-stroke patients.

### 4.2 The training intensity and mode of the EMG-based robotic intervention

The subgroup analysis suggested that the effect of the subgroup with total treatment time ≤ 1000 min was better than the subgroup with total time >1000 min (Figure 5), indicating the optimal treatment parameters to achieve the best effect of EMG-based robot therapy remains to be determined. Post-stroke fatigue is a common complication that negatively impacts patient’s rehabilitation outcomes (Duncan et al., 2012; Finsterer and Mahjoub, 2014; Maaijwee et al., 2015). One prevalent hypothesis is that physical deconditioning may contribute to fatigue following stroke (Duncan et al., 2012). One study focused on brain-computer interface (BCI) indicated that mental fatigue may also play a role in poorer BCI performance (Foong et al., 2020). Fatigue may affect patient motivation during training and interaction between human and machine, thereby hampering the efficiency of EMG-based robot. However, the relationship between post-stroke fatigue and motor training is rarely studied, and further research is needed to explore this aspect.

In the context of training mode, our results showed that the efficiency of the EMG-based robot with electrical stimulation (ES) was superior to the type without ES (Figure 6). Clinically, ES can be used to activate muscles, prevent muscle atrophy, and increase muscle strength (Hu et al., 2021; Li et al., 2022). In addition, sensory dysfunction is a common complication after stroke (Sullivan and Hedman, 2008; Tyson et al., 2008), which is related to the reduction of motor function recovery (Kusoffsky et al., 1982). ES may effectively improve sensory awareness of paralyzed muscles after stroke (Mäenpää et al., 2004), and elicit sensory feedback to the cortex during muscle contraction to facilitate motor relearning (Sujith, 2008). The sensory feedback is beneficial for motor function recovery post stroke (Sharififar et al., 2018). In the subgroup that received ES, the sensory feedback from the ES to the affected limb may be beneficial in motor function improvement. But excessive ES might impede the effect of motor training (Chae et al., 2002) and it is crucial to explore the optimal proportion of assistance from both ES and robot (Li et al., 2022). Hu et al., (Hu et al., 2011), reported that the performance of wrist tracking could be better with the 1:1 assistance from both ES and robot. But the small sample size and lack of long-term testing limited the findings of this study.

The subgroup analysis also showed that the EMG-based robot was more effective in subacute stage than chronic stage (Figure 7), which is consistent with previous studies (Mehrholz et al., 2018; Dehem et al., 2019; Dromerick et al., 2021). Early participation in rehabilitation is crucial for motor recovery as it can facilitate brain reorganization, optimize motor responsiveness and spontaneous neural plasticity, which may contribute to better rehabilitation outcomes in post-stroke patients, (Zeiler and Krakauer, 2013; Ng et al., 2015). Improved motor function in the subacute phase is more likely to generalize into activities of daily living (Flöel et al., 2014; Tomori et al., 2015).

### 4.3 Study limitations

While our subgroup analysis indicated that the effect of EMG-based robot was better in patients in the subacute stage, those who received a total treatment time of less than 1000 min, and those who received EMG-based robot combined with ES, it is important to acknowledge the limited sample size of the included studies and the potential impact on the validity of the results. Furthermore, the language restriction of our study to English-language articles may have introduced a selection bias which may also limit the generalizability of the results. The EMG-based robot requires active training and may not be suitable for patients with cognitive impairment. Future studies may explore the relationship between post-stroke fatigue and functional training to determine the best parameters of the EMG-based robot for the upper limb function, which could ultimately results in improved clinical outcomes for stroke survivors.

## 5 Conclusion

The present study provides evidence that EMG-based robot therapy is superior to conventional therapies in improving upper limb motor control, spasticity, and activity limitation in post-stroke patients. These findings suggest that EMG-based robot therapy could be a promising rehabilitation method for promoting the recovery of upper extremity function in this patient population. Further research should explore optimal parameters of EMG-based robot therapy and its long-term effects on upper limb function in post-stroke patients.

## Data Availability

The original contributions presented in the study are included in the article/supplementary materials, further inquiries can be directed to the corresponding authors.
