# Supplementary material for: Effects of EMG-based robot for upper extremity rehabilitation on post-stroke patients: a systematic review and meta-analysis
Source: Front Physiol. 2023 May 3;14:1172958. doi: 10.3389/fphys.2023.1172958 (PMC10226272; doi:10.3389/fphys.2023.1172958)
Supplement: Supplementary file 1 [file DataSheet1.PDF]

# Search Strategy

## 1. PubMed

### (1) P：stroke

**#1**

stroke (mesh) OR strokes OR cerebrovascular accident OR CVA OR CVAs OR cerebrovascular apoplexy OR brain vascular accident OR cerebrovascular stroke OR apoplexy OR cerebral stroke

### (2) I:EMG-based robot (#2 AND #3)

**#2**

robotics (mesh) OR exoskeleton device (mesh) OR orthotic devices (mesh) OR therapy,computer-assisted (mesh) OR self-help devices (mesh) OR artificial limbs (mesh) OR splints (mesh) OR remote operations OR tele robotics OR soft robotic OR exoskeleton devices OR orthotic device OR orthosis OR orthoses OR orthose OR parapodium OR parapodiums OR computer-assisted therapy OR computer-assisted therapies OR computer-assisted protocol-directed therapy OR computer-assisted protocol-directed Therapies OR assistive technology OR assistive technologies OR assistive devices OR assistive device OR limb prosthesis OR limb prostheses OR artificial limb OR artificial extremities OR artificial extremity OR artificial arm OR artificial arms OR arm prosthesis OR arm prostheses OR static orthoses OR static orthose OR static splinting OR static splint OR dynamic splints OR dynamic orthoses OR dynamic splint OR dynamic splinting OR dynamic splintings OR robotic OR robot OR bionic device OR robotic aided therapy OR robot-assisted OR robotics-assisted OR robotic device OR dynamic orthotic device OR robot-mediated therapy OR robot-supported OR rehabilitation robotics OR human-robot interaction OR robot-aided rehabilitation OR robotic rehabilitation OR splinting OR assistive technology devices OR assistive device therapy

**#3**

electromyography (MESH) OR EMG OR electromyographies OR surface electromyography OR surface electromyographies OR electromyogram OR electromyograms

## Search Strategy：#1 AND #2 AND #3

## 2. Embase

**(1) P: stroke**

**#1**

'stroke'/exp OR stroke OR 'cerebrovascular accident'/exp

**(2) I:EMG-based robot (#2 AND #3)**

**#2**

'robotics'/exp OR robotics OR 'exoskeleton device'/exp OR 'exoskeleton device' OR (('exoskeleton'/exp OR exoskeleton) AND ('device'/exp OR device)) OR 'exoskeleton (rehabilitation)'/exp OR 'orthotic devices'/exp OR 'orthotic devices' OR (orthotic AND ('devices'/exp OR devices)) OR 'orthosis'/exp OR 'therapy,computer assisted'/exp OR 'therapy,computer assisted' OR 'computer assisted therapy'/exp OR 'self help device'/exp OR 'self help device' OR 'artificial limbs'/exp OR 'artificial limbs' OR (artificial AND limbs) OR 'limb prosthesis'/exp OR 'splint'/exp OR 'robot'/exp

**#3**

'electromyography'/exp

**Search Strategy：#1 AND #2 AND #3**

## 3. Web of science

TS=(stroke OR strokes OR cerebrovascular accident OR cerebrovascular accidentS OR CVA OR CVAs OR cerebrovascular apoplexy OR brain vascular accident OR cerebrovascular stroke OR apoplexy OR cerebral stroke) AND TS=(robotics OR exoskeleton device OR orthotic devices OR therapy, computer-assisted OR self-help devices OR artificial limbs OR splints OR remote operations OR tele robotics OR soft robotic OR exoskeleton devices OR orthotic device OR orthosis OR orthoses OR orthose OR parapodium OR parapodiums OR computer-assisted therapy OR computer-assisted therapies OR computer-assisted protocol-directed therapy OR computer-assisted protocol-directed therapies OR assistive technology OR assistive technologies OR assistive devices OR assistive device OR limb prosthesis OR limb prostheses OR artificial limb OR artificial extremities OR artificial extremity OR artificial arm OR artificial arms OR arm prosthesis OR arm prostheses OR static orthoses OR static orthose OR static splinting OR static splint OR dynamic splints OR dynamic orthoses OR dynamic splint OR dynamic splinting OR dynamic splintings

OR robotic OR robot OR bionic device OR robotic aided therapy OR robot-assisted OR robotics-assisted OR robotic device OR dynamic orthotic device OR robot-mediated therapy OR robot-supported OR rehabilitation robotics OR human-robot interaction OR robot-aided rehabilitation OR robotic rehabilitation OR splinting OR assistive technology devices OR assistive device therapy) AND TS=(electromyography OR EMG OR electromyographies OR surface electromyography OR surface electromyographies OR electromyogram OR electromyograms)

## 4. Cochrane library

**(1) P: stroke**

#1

[stroke] explode all trees

**(2) I:EMG-based robot (#2 AND #3)**

**#2**

[robotics OR exoskeleton device OR orthotic devices OR therapy, computer-assisted OR self-help devices OR artificial limbs OR splints OR robot] explode all trees

**#3**

[electromyography] explode all trees

## Search Strategy：#1 AND #2 AND #3

## 5. Scpous

**(1) P: stroke**

**#1**

(stroke OR strokes OR cerebrovascular accident OR cerebrovascular accidents OR CVA OR CVAs OR cerebrovascular apoplexy OR brain vascular accident OR cerebrovascular stroke OR apoplexy OR cerebral stroke)

**(2) I:EMG-based robot (#2 AND #3)**

**#2**

(robotics OR exoskeleton device OR orthotic devices OR therapy, computer-assisted OR self-help devices OR artificial limbs OR splints OR remote operations OR tele robotics OR soft robotic OR exoskeleton devices OR orthotic device OR orthosis OR orthoses OR orthose OR parapodium OR parapodiums OR computer-assisted therapy OR computer-assisted therapies OR computer-assisted protocol-directed therapy OR computer-assisted protocol-directed therapies OR assistive technology OR assistive technologies OR assistive devices OR assistive device OR limb prosthesis OR limb prostheses OR artificial limb OR artificial extremities OR artificial extremity OR artificial arm OR artificial arms OR arm prosthesis OR arm prostheses OR static orthoses OR static orthose OR static splinting OR static splint OR dynamic splints OR dynamic orthoses OR dynamic splint OR dynamic splinting OR dynamic splintings OR robotic OR robot OR bionic device OR robotic aided therapy OR robot-assisted OR robotics-assisted OR robotic device OR dynamic orthotic device OR robot-mediated therapy OR robot-supported OR rehabilitation robotics OR human-robot interaction OR robot-aided rehabilitation OR robotic rehabilitation OR splinting OR assistive technology devices OR assistive device therapy)

**#3**

(electromyography OR EMG OR electromyographies OR surface electromyography OR surface electromyographies OR electromyogram OR electromyograms)

## Search Strategy：#1 AND #2 AND #3

# Supplementary figures

## 1. Figure A1

(a)

| | | | |
|---|---|---|---|
| Meta-regression | Number of obs | = | 10 |
| REML estimate of between-study variance | tau2 | = | 0 |
| % residual variation due to heterogeneity | I-squared_res | = | 0.00% |
| Proportion of between-study variance explained | Adj R-squared | = | 100.00% |
| Joint test for all covariates | Model F(4,5) | = | 1.91 |
| With Knapp-Hartung modification | Prob > F | = | 0.2471 |

| _ES | Coef. | Std. Err. | t | P>\|t\| | [95% Conf. | Interval] |
|---|---|---|---|---|---|---|
| subjects | .0467948 | .0333693 | 1.40 | 0.220 | -.0389838 | .1325733 |
| duration | -.0005812 | .0004624 | -1.26 | 0.264 | -.0017698 | .0006073 |
| robotmode | .4640915 | .3426191 | 1.35 | 0.234 | -.416639 | 1.344822 |
| stage | -.0001527 | .007437 | -0.02 | 0.984 | -.0192701 | .0189646 |
| _cons | .1026823 | .7064062 | 0.15 | 0.890 | -1.713193 | 1.918557 |

(b)

| | | | |
|---|---|---|---|
| Meta-regression | Number of obs | = | 7 |
| REML estimate of between-study variance | tau2 | = | 0 |
| % residual variation due to heterogeneity | I-squared_res | = | 0.00% |
| Proportion of between-study variance explained | Adj R-squared | = | 100.00% |
| Joint test for all covariates | Model F(4,2) | = | 2.56 |
| With Knapp-Hartung modification | Prob > F | = | 0.3005 |

| _ES | Coef. | Std. Err. | t | P>\|t\| | [95% Conf. | Interval] |
|---|---|---|---|---|---|---|
| subjects | -.0915896 | .041997 | -2.18 | 0.161 | -.2722881 | .089109 |
| duration | .001077 | .000966 | 1.11 | 0.381 | -.0030792 | .0052332 |
| robotmode | .244613 | .4328019 | 0.57 | 0.629 | -1.617583 | 2.106809 |
| stage | .0078712 | .0085018 | 0.93 | 0.452 | -.028709 | .0444514 |
| _cons | -.1819675 | 1.18979 | -0.15 | 0.892 | -5.301221 | 4.937286 |

(c)

| | | | |
|---|---|---|---|
| Meta-regression | Number of obs | = | 8 |
| REML estimate of between-study variance | tau2 | = | .2426 |
| % residual variation due to heterogeneity | I-squared_res | = | 61.08% |
| Proportion of between-study variance explained | Adj R-squared | = | -31.01% |
| Joint test for all covariates | Model F(5,2) | = | 0.83 |
| With Knapp-Hartung modification | Prob > F | = | 0.6249 |

| _ES | Coef. | Std. Err. | t | P>\|t\| | [95% Conf. | Interval] |
|---|---|---|---|---|---|---|
| duration | -.0006737 | .0014356 | -0.47 | 0.685 | -.0068505 | .0055032 |
| robotmode | .7883245 | .581426 | 1.36 | 0.308 | -1.71335 | 3.289999 |
| control | .3912222 | 2.061083 | 0.19 | 0.867 | -8.476901 | 9.259345 |
| stage | .0170039 | .0289327 | 0.59 | 0.616 | -.1074835 | .1414913 |
| subjects | .0090012 | .013895 | 0.65 | 0.584 | -.0507841 | .0687865 |
| _cons | -.5646757 | 1.479847 | -0.38 | 0.739 | -6.931943 | 5.802591 |

(a) Meta-regression of FMA.

(b) Meta-regression of MAS.

(c) Meta-regression of activity limitation.

## 2. Figure A2

(a)

| Study or Subgroup | Experimental Mean | Experimental SD | Experimental Total | Control Mean | Control SD | Control Total | Weight | Mean Difference IV, Random, 95% CI |
|---|---|---|---|---|---|---|---|---|
| Barker, et al. 2008 | -0.82 | 1.13767306 | 10 | 0 | 0.83 | 13 | 10.4% | -0.82 [-1.66, 0.02] |
| De Araújo, et al. 2011 | -0.17 | 0.41 | 6 | -0.17 | 0.53563047 | 6 | 19.6% | 0.00 [-0.54, 0.54] |
| Hayward, et al. 2013 | 0.5 | 0.6408588 | 4 | 0 | 0.19 | 4 | 0.0% | 0.50 [-0.16, 1.16] |
| Hu, et al. 2008 | -0.41 | 0.90504144 | 5 | -0.05 | 0.94978945 | 5 | 6.1% | -0.36 [-1.51, 0.79] |
| Hu, et al.2009 | -0.6 | 0.65574385 | 15 | 0.1 | 0.36055513 | 12 | 28.0% | -0.70 [-1.09, -0.31] |
| Huang, et al.2020 | -0.77 | 0.87469995 | 15 | -0.24 | 0.79504717 | 15 | 17.2% | -0.53 [-1.13, 0.07] |
| Qian, et al.2017 | -0.5 | 0.87 | 14 | 0.5 | 0.52848841 | 10 | 18.7% | -1.00 [-1.56, -0.44] |
| **Total (95% CI)** | | | **65** | | | **61** | **100.0%** | **-0.58 [-0.88, -0.28]** |

Heterogeneity: Tau² = 0.05; Chi² = 7.43, df = 5 (P = 0.19); I² = 33%

Test for overall effect: Z = 3.77 (P = 0.0002)

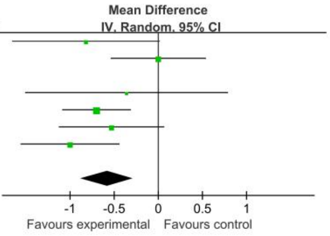


(b)

| Study or Subgroup | Experimental Mean | Experimental SD | Experimental Total | Control Mean | Control SD | Control Total | Weight | Std. Mean Difference IV, Fixed, 95% CI |
|---|---|---|---|---|---|---|---|---|
| Ambrosini, et al. 2021 | 17.9 | 17.69943502 | 36 | 8.5 | 18.59220267 | 36 | 33.9% | 0.51 [0.04, 0.98] |
| Chen, et al. 2022 | 1.5 | 9.13681016 | 14 | 3.6 | 11.34144171 | 10 | 0.0% | -0.20 [-1.01, 0.61] |
| Hu, et al. 2009 | 1.1 | 11.41183596 | 15 | 0.4 | 11.4660368 | 12 | 13.0% | 0.06 [-0.70, 0.82] |
| Huang, et al. 2020 | 13.47 | 12.38697703 | 15 | -4.86 | 12.69086679 | 15 | 11.3% | 1.42 [0.61, 2.24] |
| Immick, et al. 2018 | 26.1 | 17.7163766 | 19 | 5.2 | 19.29429968 | 20 | 16.3% | 1.10 [0.43, 1.78] |
| Perini, et al. 2019 | 2.4 | 17.4502149 | 6 | 1.2 | 18.58090418 | 5 | 5.3% | 0.06 [-1.13, 1.25] |
| Perini, et al. 2021 | 1.9 | 14.54338338 | 9 | 0.8 | 13.75790682 | 9 | 8.8% | 0.07 [-0.85, 1.00] |
| Qian, et al. 2017 | 13.5 | 11.95 | 14 | 12.2 | 11.18 | 10 | 11.4% | 0.11 [-0.70, 0.92] |
| **Total (95% CI)** | | | **114** | | | **107** | **100.0%** | **0.54 [0.27, 0.82]** |

Heterogeneity: Chi² = 11.41, df = 6 (P = 0.08); I² = 47%

Test for overall effect: Z = 3.90 (P < 0.0001)

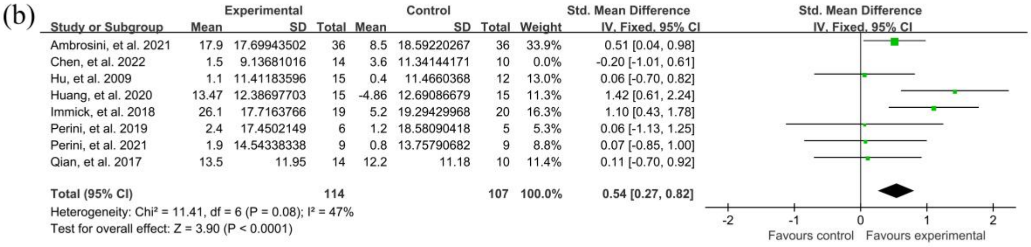


(a) The outcome of MAS.

(b) The outcome of activity limitation.
